# The direct way may not be the best way: Children with ADHD and their understanding of self-presentation in social interactions

**DOI:** 10.1080/17405629.2015.1051960

**Published:** 2015-06-22

**Authors:** Daniela Kloo, Winfried Kain

**Affiliations:** ^a^ Department of Psychology, University of Salzburg, Hellbrunnerstr. 34, A-5020Salzburg, Austria

**Keywords:** Attention-deficit/hyperactivity disorder, Social skills, Self-presentation

## Abstract

Knowledge and use of self-presentational tactics is an important social skill. We examined understanding of the function of three different self-presentational tactics (self-promotion, ingratiation and blasting) in 11 8–12-year-old boys with attention-deficit/hyperactivity disorder (ADHD) and 11 matched comparison children. Children were given six different self-presentation stories, two for each one of the three different tactics. After each story, they were asked to evaluate the effects of the self-presentational tactic used. Children with ADHD rated self-promotion and blasting as more positive and more effective—and ingratiation as less positive and less effective—than children in the control group. This implicates that children with ADHD prefer simple and direct self-presentational strategies (like self-promotion), and, therefore, may not as easily understand more subtle strategies (like ingratiation). They also seem to be more inclined to use negatively connoted strategies (like blasting). We suggest that this limited understanding of self-presentational strategies in children with ADHD may explain some of their problems in social interactions. Therefore, social skill interventions in children with ADHD should incorporate elements focusing on use and understanding of different self-presentational strategies.

Attention-deficit/hyperactivity disorder (ADHD) is a highly prevalent neurobehavioural disorder that occurs in 3–7% of children. The aetiology of ADHD is believed to be multifactorial including genetic, biological and neuropsychological factors. ADHD comprises two separate domains of symptoms: inattention and hyperactivity–impulsivity. Based on these two domains, the Diagnostic and Statistical Manual of Mental Disorders (DSM-IV; American Psychiatric Association [APA], 2000) distinguishes between three subtypes of ADHD: a predominantly inattentive subtype (ADHD-I), a predominantly hyperactive/impulsive subtype (ADHD-H) and a combined subtype, though empirical evidence for the validity of these three subtypes is scarce (Willcutt et al., 2012).

ADHD can lead to various impairments in school performance, family life and social interactions. Children with ADHD often have problematic peer relationships with up to 80% of children with ADHD experiencing peer rejection (see Hoza, 2007, for a review). For example, children with ADHD are less well liked and more often rejected by their peers than comparison children (Hoza et al., 2005). Furthermore, they are not chosen as often to be best friends or partners in activities (Goldstein & Kennemer, 2009).

Reasons for these peer relationship problems may lie in the core symptoms of ADHD: inattention may limit social learning and attention to social cues. And the impulsive and hyperactive behaviour of children with ADHD may make them highly aversive to peers. Moreover, in general, children with ADHD show deficits in social skills such as self-evaluation, self-monitoring and appropriate response to social cues (Hoza, 2007).

One important social skill is our knowledge and use of self-presentational tactics in order to actively influence the impressions we convey to others (impression management; Goffman, 1959; Leary & Kowalski, 1990). In this study, we focus on the strategies of self-promotion, ingratiation and blasting. For example, people use self-promotion (e.g., self-enhancing comments, claiming competence) in order to be perceived as a competent, skilled person. People also often engage in ingratiation (e.g., compliments, favours) in order to be liked by another person. Blasting (presenting negative information about a rival) is used as an indirect form of self-enhancement (using devaluating information about a rival in order to be perceived as “better than the rival”).

We conceptualize self-promotion as a direct strategy (i.e., focusing on self) and ingratiation and blasting as indirect self-presentational strategies (i.e., focusing on others): self-promotion is directly linked to the desired social outcome (being perceived as, for example, capable). By contrast, the self-enhancing function of blasting works indirectly (via the devaluation of potential rivals). Also, when people use ingratiation, the desired social outcome (being perceived as likable) is achieved indirectly through a positive social evaluation (due to other-enhancing comments). In addition, self-promotion and blasting can be viewed as rather obvious self-presentational tactics because of their apparent self-enhancing function. By contrast, ingratiation can be considered as a rather sophisticated and less obvious tactic.

Other well-known self-presentational tactics used to convey a positive self-image include modesty (downplaying one's achievements to evoke a positive social evaluation), excuses (denying responsibility for negative events) and disclaimers (explain negative events before they occur).

In typically developing children, use and understanding of impression management tactics (like modesty, self-promotion and ingratiation) develop during middle childhood at around 8 years of age (e.g., Aloise-Young, 1993; Banerjee, 2000, 2002; Benne & Yeeles, 1990; Watling & Banerjee, 2007a, 2007b). In an early study by Benne and Yeeles (1990), 64 8-year-olds and 56 11-year-olds were presented with stories depicting a protagonist who tries to get picked as a new team member using either a self-promotion (“I'm very good”) or an ingratiation (“I bet you're very good”) statement. It was found that understanding of self-presentation considerably improved from 8 to 11 years of age. Similarly, Watling and Banerjee (2007a) provided evidence that children understand the different processes involved in self-promotion and ingratiation between 6 and 11 years of age. Specifically, ingratiation was rated as leading to more positive social evaluations than self-promotion, whereas self-promotion was rated as having a more concrete, instrumental function. Furthermore, at approximately 8 years of age, children are able to provide interpersonal explanations for self-presentational acts (Banerjee & Yuill, 1999). The social value of a modest self-presentation is also understood by approximately 8 years of age (Watling & Banerjee, 2007b; see also Banerjee, 2000; Yoshida, Kojo, & Kaku, 1982).

Previous research has also shown that, in middle childhood, children understand that self-presentational tactics may be modified in front of different audiences. For example, Banerjee (2002) found that 10- to 11-year-olds, but not younger children, judged that a new child should emphasize academic competence to an adult audience and athletic competence to a peer audience. Better audience differentiation was associated with more reciprocal nominations as a playmate (see also Watling & Banerjee, 2007a, for evidence that understanding of self-presentation tactics is related to peer preference scores, at least in boys). This suggests that understanding of and using self-presentational strategies are related to children's social success (see also Juvonen, 1996).

Given the fact that children with ADHD have problems with social interactions (Hoza, 2007) and that knowledge and use of self-presentational strategies is an important social skill, in the present study, we investigated the evaluation of different self-presentational tactics in children with ADHD and typically developing peers. To this end, we used three self-presentational tactics: self-promotion, ingratiation and blasting.

Children were given different stories, inspired by Watling and Banerjee (2007a) and Benne and Yeeles (1990), about a hypothetical story character, who tries to become part of a team and uses either an ingratiating, or a self-promoting, or a blasting statement. After the story, children were asked about the motives behind and consequences of the protagonist's self-presentational statement.

The strategies of self-promotion and ingratiation have already been used in previous research (e.g., Benne & Yeeles, 1990; Watling & Banerjee, 2007a). In these studies, it has been found that children's understanding of these two tactics improves during primary school age. However, to the best of our knowledge, there is no systematic investigation of use and understanding of blasting in children. Blasting refers to the presentation of negative, devaluating information about a rival in order to enhance one's own image. It has been conceptualized as an indirect tactic of self-presentation (Cialdini & Richardson, 1980) and is seen as a core assertive self-presentational tactic by Lee, Quigley, Nesler, Corbett, and Tedeschi (1999).

We hypothesized that, due to their impulsivity and working memory impairments (Martinussen, Hayden, Hogg-Johnson, & Tannock, 2005; Raiker, Rapport, Kofler, & Sarver, 2012), children with ADHD would, in contrast to typically developing peers, rate the most direct (and most obvious) strategy of self-promotion as most effective and would show less understanding of the indirect strategies of ingratiation and blasting. We also expected that typically developing children would rate blasting as a rather negative and ineffective strategy. In general, blasting is not seen as socially desirable, because it could hurt other people's feelings, and, therefore, may create a negative impression of the protagonist. By contrast, children with ADHD may not rate blasting as that ineffective, because they are less likely to understand and follow social rules.

## METHOD

### Participants

A total of 22 German-speaking children aged between 8 and 12 years of age (*M* = 119.73 months, *SD* = 17.47 months, range 96–152 months) took part in this study. Eleven boys with ADHD were recruited through a practice of a child psychiatrist, and 11 boys without ADHD were recruited through after-school child-care centres. All participants with ADHD met DSM-IV criteria for ADHD (APA, 2000); they ranged in age from 99 to 136 months (*M* = 118.55 months, *SD* = 11.51 months). The boys without ADHD were between 96 and 152 months (*M* = 120.91 months, *SD* = 22.47 months). The two groups did not differ in age, *t*(20) = .31, *p* = .76. Parents received full information about the study. Informed parental consent was obtained for all children who participated in the experiment. Boys in the age-matched control group were excluded if they showed clinically significant scores on the ADHD-Rating Scale-IV (DuPaul, Power, Anastopoulos, & Reid, 1998). All children were unmedicated on the day of testing.

### Procedure

Children were tested individually in two 45-min sessions, about 1 week apart, as part of a larger study. Each child received six different self-presentation stories (three stories per session). The self-presentation stories had a similar format as the ones used by Watling and Banerjee (2007a; see also Benne & Yeeles, 1990). Children were presented with six illustrated stories (see example in Appendix), two for each one of the three different tactics: ingratiation (making oneself seem likeable), self-promotion (making oneself seem competent) and blasting (devaluating a rival).

Story content and the self-presentation tactic statement used were counterbalanced. Stories were presented in a fixed order. However, the self-presentation statement used in each story varied between children. Children always heard stories with protagonists matching their own gender, that is, all protagonists were boys.

The protagonist was a child, who has just moved to a new school and wanted another character to select him for some team activity. To this end, the protagonist uttered either an ingratiating statement (e.g., “Max, I bet you are a very fast runner. You can probably handle the ball better and run faster than anyone in this school”), or a self-promotion statement (e.g., “Max, I am a very fast runner. I can handle the ball better and run faster than anyone in this school”), or a blasting statement (e.g., “Max, I think all the other substitutes are rather slow runners and cannot handle the ball very well”).

After each story, participants had to evaluate the self-presentational tactic used by the protagonist (self-promotion, ingratiation, or blasting). In line with Watling and Banerjee (2007a; see also Benne & Yeeles, 1990), children were asked to rate: (1) how likely it is that the protagonist will be selected for the team activity (*inclusion judgement*; rated on a four-point scale: definitely will not, probably will not, probably will and definitely will, scored 0–3); and (2) how nice the other character would think the protagonist was (*character judgement*; rated on a four-point scale: not at all nice, a little bit nice, quite nice and very nice, scored 0–3).

For each pair of stories using the same self-presentation tactic (self-promotion, ingratiation and blasting), the inclusion and character judgement scores were summed across the two stories. Therefore, for each self-presentation tactic, children received a score from 0 to 6 for the inclusion judgement as well as for the character judgement. Higher scores indicated a greater likelihood of being selected for the team or being judged as nice.

In addition, children were asked to justify why the protagonist said what he did (*justification*). Children's answers were coded into one of five categories, following the coding scheme of Watling and Banerjee (2007a, p. 762; see also Banerjee, 2000):

1. *Social evaluation*: Reference to motivation to manipulate what the other person thinks of the protagonist (e.g., “So he will think that he is a nice person”).
2. *Social outcome*: Reference to a concrete goal or purpose of the protagonist's statement (e.g., “So he will pick him for the team”).
3. *Others' feelings*: Reference to others' feelings (e.g., “So that he will feel happy”)
4. *Truth*: Reference to the “true” state of affairs, that is, characterizing the protagonist's statement as a description of reality (e.g., “Because he is good at sports”)
5. *Residual*: Any other response (e.g., nonsense justifications and “don't know” responses)

All 132 justifications were coded by two independent raters. There were only seven disagreements, which were resolved through discussion. The number of justifications of each category was counted across all six stories (possible range of 0–6 for each category).

## RESULTS

All analyses were realized on the SPSS program, version 22.0, with a 5% level of significance. Owing to the small sample size, *t*-tests with re-sampling methods were used because of their robustness against small samples. By bootstrap analysis with 1000 replicates, the bounds for the 95% confidence interval were determined. Significance was determined if the confidence interval did not include zero.

Table 1 shows the mean inclusion and character ratings in the two groups for the three self-presentational tactics.

**TABLE 1:** Mean scores (*SD*) for the inclusion and character judgements of the three self-presentational tactics in the ADHD and control group

	Inclusion		Character	
Tactic	ADHD	Control	ADHD	Control
Ingratiation	1.90 (.77)	2.45 (.41)	2.36 (.59)	2.72 (.34)
Self-promotion	1.95 (.82)	1.18 (.60)	1.50 (.74)	.59 (.44)
Blasting	1.64 (.55)	.36 (.39)	1.00 (.55)	.09 (.20)

*Note*: Maximum score = 3.

Performance on the *inclusion question* was analysed with a 3 × 2 mixed design analysis of covariance (ANCOVA), with the self-presentational tactic (self-promotion, ingratiation or blasting) as the within-participants factor, group (ADHD vs. control) as a between-participants factor and age as a covariate. Age was used as a covariate, because we did not expect age differences based on previous research; Watling and Banerjee (2007a, Exp. 1) found only significant differences between their youngest age group (6- to 7-year-olds) and older children.

This ANCOVA revealed a significant main effect of group, *F*(1, 19) = 10.31, MSE = .391, *p* = .005, with children in the ADHD group offering higher inclusion judgements than children in the control group. The main effect was further qualified by a significant group × tactic interaction, *F*(2, 38) = 12.27, MSE = .389, *p* < .001. There was no significant main effect of tactic. Moreover, there was no significant effect of or interaction with age.

The significant interaction between group and tactic was due to a different evaluation of all three tactics in the two experimental groups: in terms of the likelihood of being included in the team, ingratiation was rated as more effective by the control group (*M* = 2.45, *SD* = .41) than by the ADHD group (*M* = 1.90, *SD* = .77), *t*(20) = 2.07, *p* = .052, *d* = .89. By contrast, self-promotion was rated as more effective by the ADHD group (*M* = 1.95, *SD* = .82) than by the control group (*M* = 1.18, *SD* = .60), *t*(20) = 2.52, *p* < .05, *d* = 1.07. In fact, control children viewed ingratiation (*M* = 2.45, *SD* = .41) as more effective than self-promotion (*M* = 1.18, *SD* = .60), *t*(10) = 6.17, *p* < .001, *d* = 2.47, whereas children with ADHD did not differentially evaluate these two strategies, *t*(10) = .12, *p*>.05. The sharpest group difference was evident on the blasting tactic: children with ADHD rated this strategy as far more effective (*M* = 1.64, *SD* = .55) than children in the control group (*M* = .36, *SD* = .39), *t*(20) = 6.23, *p* < .001, *d* = 2.68. This suggests that children with ADHD evaluate the different interpersonal consequences of different self-presentational tactics in a different way.

Performance on the *character question* was analysed with a parallel ANCOVA. Again, there was a significant main effect of group, *F*(1, 19) = 12.29, MSE = .304, *p* = .002, with children in the ADHD group giving higher character judgements than children in the control group. There was also a significant main effect of tactic, *F*(2, 38) = 4.08, MSE = .244, *p* < .05, with children in both groups giving the highest character judgements in the ingratiation stories and the lowest character judgements in the blasting stories. In addition, there was a significant group × tactic interaction, *F*(2, 38) = 12.46, MSE = .244, *p* < .001. There was no significant effect of or interaction with age.

Again, post hoc *t*-tests showed significant (or approaching significant) differences between the two experimental groups for each self-presentational tactic on the character judgement. Similar to performance on the inclusion question, ingratiation was rated more positively (i.e., resulting in higher character judgements) by the control group than by the ADHD group. However, this difference was not significant, *t*(20) = 1.75, *p* < .10, *d* = .75. By contrast, self-promotion, *t*(20) = 3.50, *p* = .002, *d* = 1.49, and blasting, *t*(20) = 5.16, *p* < .001, *d* = 2.20, were rated more positively by the ADHD group than by the control group (Table 1).

Children's justifications of the protagonist's self-presentational statement also varied in the two experimental groups (Table 2). A multivariate ANCOVA with age as a covariate was performed to determine the effects of group (ADHD vs. control) on the number of justifications given per category (possible range 0–6, summed across the six scenarios). Group differences were found for the number of social evaluation justifications, *F*(1, 19) = 80.22, MSE = .81, *p* < .001 (ADHD: *M* = .64, *SD* = 1.03; control: *M* = 4.09, *SD* = .70), for the number of social outcome justifications, *F*(1, 19) = 13.36, MSE = 1.77, *p* = .002 (ADHD: *M* = 3.27, *SD* = 1.62; control: *M* = 1.18, *SD* = .87) and for the number of others' feelings justifications given, *F*(1, 19) = 6.13, MSE = .13, *p* = .023 (ADHD: *M* = .00, *SD* = .00; control: *M* = .36, *SD* = .50). That is, children with ADHD offered more social outcome justifications and fewer social evaluation justifications than children in the control group. In contrast to the control group, they did not refer to others' feelings. However, four children with ADHD, but none in the control group, referred to “truth” as an explanation for the protagonist's behaviour; though this effect was not statistically significant.

**TABLE 2:** Numbers (%) of children in the ADHD and control groups giving at least one justification in the respective category (social evaluation, social outcome, others' feeling, truth or residual)

	Social evaluation	Social outcome	Others' feeling	Truth	Residual
ADHD (*n* = 11)	4 (36)	10 (91)	0	4 (36)	5 (45)
Control (*n* = 11)	11 (100)	8 (73)	4 (36)	0	3 (27)

## DISCUSSION

The main goal of this study was to examine how children with ADHD evaluate different self-presentational tactics (self-promotion, ingratiation and blasting), and it was found that children with ADHD show a distinctive pattern of self-presentational understanding. They consistently rated self-promotion and blasting as more positive—and ingratiation as less positive -—than children in the control group. This effect was evident on an inclusion judgement (“How likely is it that the protagonist will be selected for the team?”) as well as on a character judgement (“How nice will Max think that the protagonist is?”). Nevertheless, similar to Watling and Banerjee (2007a, Exp. 1), both the control group and children with ADHD rated ingratiation as resulting in the highest character judgements. Generally, children with ADHD gave higher character judgements than children in the control group.

In a similar vein, when asked to justify the protagonist's statement, children with ADHD used less sophisticated explanations. While children in the control group offered mainly social evaluation justifications, children with ADHD gave mainly social outcome justifications for the different strategies, suggesting that they focus on immediate, but superficial effects of self-presentational behaviour. Owing to their working memory and inhibition problems, children with ADHD may not thoroughly reflect on possible effects, and may instead respond impulsively by selecting any plausible strategy that could work. This may prevent them from understanding how different self-presentational tactics create different impressions. In other words, children with ADHD may simply take each self-presentational strategy as one of the various, but equally plausible, means for obtaining a desired goal (without taking into consideration that different strategies yield different social evaluations, e.g., “being a nice or nasty person”).

In sum, our study shows that children with ADHD rate simple, obvious tactics (like self-promotion and blasting) as more effective than control children; children in the control group viewed these tactics, especially blasting, as quite ineffective strategies. This differential evaluation of self-presentational strategies may be a consequence of the working memory impairments (e.g., Martinussen et al., 2005) in children with ADHD, because limited memory capacity may prevent them from easily understanding more complex, sophisticated self-presentation strategies. For example, the tactic of self-promotion is a simple way of self-enhancement (by highlighting one's qualities or mentioning personal achievements). By contrast, the motivation for using ingratiation is less apparent: by highlighting the other person's qualities, the ingratiator will be viewed as more likeable.

Owing to this, children with ADHD may also over-estimate the benefit of obvious self-presentational tactics in their daily social interactions, making them less socially effective in their peer interactions (e.g., Hoza, 2007). Indeed, there is evidence that ingratiation is more effective than self-promotion: Judge and Bretz (1994) found that ingratiation is positively related to career success, whereas self-promotion has a negative effect on career success.

Importantly, in general, and the more so in children with ADHD, we have to distinguish between social skill deficits and performance deficits. That is, children with ADHD might have the relevant social skills, but are unable to apply them (cf. Gresham, Sugai, & Horner, 2001; Nilsen & Fecica, 2011). In fact, a number of studies suggest that children with ADHD are not impaired on social cognition or theory of mind tasks compared to their typically developing peers (e.g., Charman, Carroll, & Sturge, 2001; Perner, Kain, & Barchfeld, 2002), but show impairments in executive functions like working memory (e.g., Martinussen et al., 2005) or inhibition (e.g., Nigg, 2001). This could suggest, as noted above, that, in the current study, children with ADHD rated the most direct and most obvious strategy of self-promotion as most effective simply due to their working memory and inhibition impairments. That is, children with ADHD may have problems to step back from the immediate situation (due to inhibitory weaknesses) and to reflect on possible causes of a person's behaviour (due to working memory limitations). This may lead to a focus on simple self-presentational tactics.

In future studies, it may be important to distinguish between social knowledge deficits and social performance deficits in children with ADHD. For example, when investigating use and understanding of self-presentational strategies, children's judgement of a protagonist's behaviour in a fictional story (as in the present study) should be combined with observational measures of self-presentational behaviour in everyday peer interactions; as the latter may be more sensitive to performance deficits.

Further research might also focus on use and understanding of self-presentational tactics in the different ADHD subtypes, because, as suggested by Wheeler and Carlson (1994; see also Wheeler Maedgen & Carlson, 2000), skill (knowledge) deficits and performance deficits may play different roles in ADHD subtypes. For example, children with ADHD-H may possess the relevant social skills, but have difficulties to apply them due to their inhibition deficits and problems in emotional regulation. By contrast, children with ADHD-I may not even possess the relevant social skills, because their withdrawal from social interactions, their social passivity and their lack of concentration impair the acquisition of social skills.

Concerning treatment approaches in children with ADHD, our findings tentatively suggest that interventions designed to improve the understanding of more subtle and indirect self-presentational tactics should be pursued. The more so as childhood social skill deficiencies and peer-relationship difficulties have been linked to later adjustment problems, delinquency and psychopathology (e.g., Cowen, Pederson, Babigian, Izzo, & Trost, 1973; Kupersmidt & Coie, 1990; Parker & Asher, 1987), and children with ADHD are often rated as unpopular by their peers (e.g., Carlson, Lahey, Frame, Walker, & Hynd, 1987; Lahey, Schaughency, Strauss, & Frame, 1984). Though, it should be noted that, unfortunately, in general, social skill trainings are not particularly effective in improving social competence and peer relationships of children with ADHD (e.g., Antshel & Remer, 2003; Hoza, 2007). Therefore, a comprehensive training approach should also convey the importance of self-presentation for social interactions and train self-presentational tactics in relevant social contexts—in addition to general social skills and self-control training.

## Appendix

This is Max. He is the goal keeper in the football team, and everyone in the class likes him. One day, Max's classmate told him that a boy on his football team was sick. Therefore, Max would need to find another boy for his team for the football game the next day. Thomas, who was the new boy in the class, heard the conversation between Max and his classmate. Thomas went up to Max and said, “You know Max, I am a very fast runner. I can handle the ball very well and I can run faster than anyone in this school” (self-promotion); *or* “You know Max. I bet you are a very fast runner. You look like you can handle the ball very well and you can probably run faster than anyone in this school” (ingratiation); *or* “You know Max. All other possible substitutes are rather slow runners. And they cannot handle the ball very well” (blasting)
